# Evaluation of Maternal Serum sHLA-G Levels for Trisomy 18 Fetuses Screening at Second Trimester

**DOI:** 10.3389/fgene.2020.497264

**Published:** 2021-01-26

**Authors:** Danping Xu, Yiyang Zhu, Lanfang Li, Yingping Xu, Weihua Yan, Meizhen Dai, Linghong Gan

**Affiliations:** ^1^Reproductive Center, Taizhou Hospital of Zhejiang Province, Wezhou Medical University, Wenzhou, China; ^2^Medical Research Center, Taizhou Hospital of Zhejiang Province, Wezhou Medical University, Wenzhou, China

**Keywords:** human leukocyte antigen-G, soluble HLA-G, pregnancy, fetus, chromsome, trisomy

## Abstract

Human leukocyte antigen-G (HLA-G) has been widely acknowledged to play critical roles in fetal-maternal maintenance. However, the significance of using maternal serum sHLA-G to detect prenatal chromosomal abnormality has not been investigated. In China, prenatal screening using maternal α-fetoprotein (AFP), unconjugated estriol (uE3), and free β subunit human chorionic gonadotropin (β-hCG) in the second trimester has been widely applied. In this study, we evaluated the use of sHLA-G as a screening marker, compared with traditional second trimester prenatal screening. Serum samples from 1,019 singleton women in their second trimester were assessed. Among them, 139 infants were confirmed with trisomy 21 (T21) by karyotyping, 83 were confirmed with trisomy 18 (T18), and the remaining 797 infants had no abnormalities. The sHLA-G levels in maternal sera were significantly lower in pregnant women with T18 fetuses (median: 47.8 U/ml, range: 9.8–234.2 U/ml) and significantly higher in those with T21 fetuses (median: 125.7 U/ml, range: 28.7–831.7 U/ml), compared with the normal controls (median: 106.3 U/ml, range: 50.5–1136.4 U/ml) (*p* < 0.001). The risk values of the screening of T21 or T18 fetuses were assessed using mean and standard deviation log_10_ analyte multiples of median (MoM) which showed that the predictive values of sHLA-G were the same as free β-hCG, and superior to AFP and uE3 for T18 screening. Logistic regression analysis revealed that sHLA-G MoM was the highest risk factor associated with pregnant women carrying T18 fetuses [Exp(B): 171.26, 95% CI: 36.30–807.97, *p* < 0.001]. Receiver operating characteristic (ROC) analysis revealed that the area under ROC curve for sHLA-G MoM was 0.915 (95% CI, 0.871–0.959, *p* < 0.001), for AFP MoM was 0.796 (95% CI, 0.730–0.861, *p* < 0.001), for free β-hCG MoM was 0.881 (95% CI, 0.829–0.934, *p* < 0.001), and for uE3 MoM was 0.876 (95% CI, 0.828–0.923, *p* < 0.001) in the T18 group. sHLA-G MoM demonstrated the best sensitivity and negative predictive value. For the first time, our findings reveal that sHLA-G is a better second trimester screening marker for the detection of T18 fetuses and the combined application of sHLA-G with AFP, free β-hCG, and uE3 could improve clinical screening for T18 fetuses.

## Introduction

China is a large country, with a population size of more than 1.3 billion people. With an aging population in mind, the Chinese government canceled the birth control policy on October 29, 2015. The number of pregnant women increased sharply. Trisomy 18 syndrome (T18), also known as Edwards syndrome, is one of the most common aneuploidies diagnosed in the prenatal period. Most T18 fetuses are miscarried, so the overall prevalence in China is difficult to estimate, but the prevalence of newborn babies with T18 is 1 in 5,000 live births. Although non-invasive prenatal testing (NIPT) could be used to screen for T18 in the early stages of pregnancy, its cost meant it could not be made widely available. In China, combined serological screening in the first and second trimesters had been widely introduced in recent years and there were also some regions or individuals who simply chose the second trimester screening. Therefore, it is important to improve the detection rate and to reduce the false-positive rate for diagnosing this condition.

Human leukocyte antigen-G (HLA-G) was cloned and identified by Geraghty in 1987 and its expression was first observed in human trophoblasts by Kovats et al. (1990). Since then, a large number of studies have explored the biological roles and clinical significance of HLA-G in the arena of reproductive immunology (Ferreira et al., 2017). At least seven isoforms of HLA-G have now been well-defined, including four membrane-bound (mHLA-G; HLA-G1 to -G4) and three soluble (sHLA-G; HLA-G5 to -G7) isoforms; and additional novel isoforms have been proposed as a result of alternative splicing of the primary transcripts (Tronik-Le Roux et al., 2017; Lin and Yan, 2018). Both mHLA-G and sHLA-G have comprehensive immune suppression functions including inhibition of T cells, natural killer (NK) cell cytotoxicity (Huang et al., 2009; Chen et al., 2013), and stimulating proliferation of regulatory cells such as regulatory T cells (Tregs) and myeloid-derived suppressor cells (MDSCs) (Tilburgs et al., 2015; Kostlin et al., 2017). The underlying mechanisms involve interactions between the HLA-Gs and their receptors, such as the immune inhibitory receptor immunoglobulin-like transcripts (ILT)2 and ILT4, which are expressed on various immune cells (Nowak et al., 2017; Lin and Yan, 2018). The concept that HLA-G plays critical roles in semi-allograft maternal-fetal immune tolerance has been well-established (Ferreira et al., 2017; Persson et al., 2017). It is worth noting that a recent study reported that crosstalk between HLA-G and ILT2 is able to stimulate the CD49a^+^Eomesodermin^+^uterine NK (uNK) secretion of growth-promoting factors (Fu et al., 2017), which contribute to fetal growth. The authors also found that decreased growth-promoting factors (GPFs) secretion was able to impair fetal development and resulted in fetal growth restriction.

In the context of pregnancy complications, decreased maternal peripheral sHLA-G levels have been observed in cases of recurrent miscarriage, pre-eclampsia, and intrauterine growth restriction (Laskowska et al., 2012; He et al., 2016; Zidi et al., 2016). The interaction of HLA-G with the receptors expressed on uNK cells (the dominant leukocyte population at the maternal-fetal interface) can play essential roles in a successful pregnancy, including spiral artery remodeling, trophoblast invasion and control of viral infection (Yan et al., 2007; van Beekhuizen et al., 2010; Tilburgs et al., 2015).

However, whether maternal peripheral blood sHLA-G levels can be used to diagnose prenatal chromosomal abnormality remains unknown. In this study, the levels of sHLA-G in maternal sera were determined using ELISAs on serum samples from pregnant women whose fetuses or babies presented with normal (797 women) or abnormal karyotypes (222 women). The relationship between maternal sHLA-G levels and the status of fetal karyotypes was evaluated to determine the potential for sHLA-G in the second trimester as a tool for prenatal screening.

## Subjects and Methods

### Study Population

**Figure 1:**
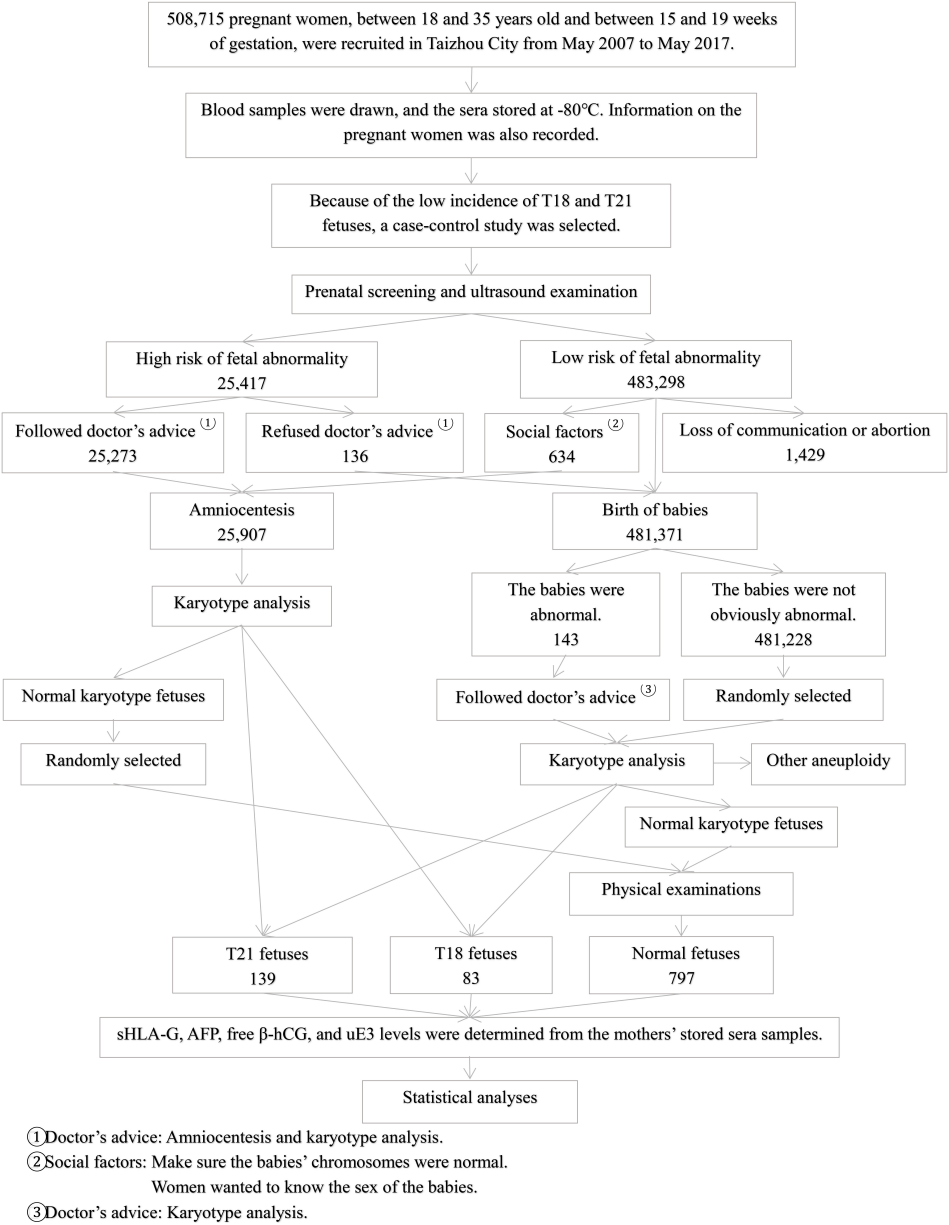
Flow chart of the participant selection method.

We retrospectively reviewed pregnant women who received second trimester prenatal screening from May 2007 to May 2017 in our Taizhou prenatal screening and diagnostics center, Taizhou Hospital of Zhejiang Province, China. During this period, 508,715 pregnant women received prenatal screening and all of them were between the ages of 18 and 35, with gestational weeks ranging from 15 to 19 weeks. Because of the low incidence of T18 and T21 fetuses, a case-control study was selected. A sample of cases and controls were selected from a cohort of 508,715 pregnant women. The participant selection method is shown in Figure 1. Risk estimation was based on the levels of the prenatal serum markers α-fetoprotein (AFP), free β subunit human chorionic gonadotropin (β-HCG), and unconjugated estriol (uE3). Within this cohort, 25,417 high-risk cases and 634 low-risk cases underwent invasive prenatal diagnosis with amniocentesis and amniotic fluid karyotype analysis. In addition, chromosome karyotypes for peripheral blood or umbilical cord blood were analyzed from 594 babies and dead fetuses. In large hospitals in Zhejiang province, karyotype analysis and the genetic testing for deafness have become necessary tests for newborns in recent years. Therefore, all newborns will participate in peripheral blood karyotype analysis in the future.

In China, children under 1 year old had many important physical examinations. The examinations included measurement of height, weight, and head circumference, the fontanelle size, the number of baby teeth, vision development, and genital development. All pregnant women who received prenatal screening in our center were followed up with 1 year after their babies' birth. In total, second trimester screening for T18 and T21 had a detection rate of 66 of 83 T18 cases (79.5%) and 105 of 139 T21 cases (75.5%) for a 5% false-positive rate, respectively. We found 222 fetuses or babies with abnormal karyotypes, including 139 with trisomy 21 (T21) and 83 with trisomy 18 (T18). These mothers were treated as experimental groups. In contrast, the mothers of fetuses or babies with normal karyotypes were treated as controls. Various problems might occur during pregnancy, such as a high risk of abnormality, polyhydramnios, oligohydramnios, bright light in the fetal ventricle, single umbilical artery. These problems do not affect the health of children after the exclusion of abnormal karyotypes. Therefore, these factors were included in the control group. All fetuses or babies were singletons who were naturally conceived. We excluded all pregnant women whose babies presented with problems at the follow up examinations, who had multiple babies, or who conceived by *in vitro* fertilization.

The remaining subjects were divided into two groups: amniocentesis and non-puncture groups. Karyotype analysis was performed on the amniotic fluid from pregnant women in the amniocentesis group, which was further divided into two groups based on the reason for puncture: high risk of abnormality due to prenatal screening and other reasons. The total control group was randomly selected from among these participants. In the control group selected from the non-puncture group, peripheral blood was collected from the children (within 2 years old) for karyotype analysis. Subjects with karyotype abnormalities were excluded and 797 cases were finally selected to comprise the normal control group. All of the children who were examined by ultrasound were normal in the normal control group.

In the control group selected from the amniocentesis group, 297 pregnant women were at high risk of abnormality by prenatal screening, 115 pregnant women were diagnosed with abnormality by ultrasound (48 cases with polyhydramnios, 11 cases with oligohydramnios, 34 cases with bright light in the fetal ventricle, and 22 cases with single umbilical artery) and 49 women had normal pregnancies. The 49 pregnant women were urged to take amniotic fluid for chromosome examination due to social factors.

This study was approved by the Ethics Committee of Taizhou Hospital of Zhejiang Province, and all patients provided informed consent after prenatal screening, in accordance with the Declaration of Helsinki. Both karyotype analysis and physical examination were the screening gold standards in this study and were performed on all fetuses or babies. We applied the double-blind method to sample selection and detection. All of the sera were stored in a refrigerator at −80°C. Table 1 summarizes the subject information of the pregnant women.

**Table 1:** Maternal age, weight, and sHLA-G level of the study population.

**Grouping**	***N***	**Median weight (range)**	**Median age (range)**	**Median sHLA-G (range)**
Pregnant women with T21 fetuses	139	54.5 (36.8–93.0)	28.65 (18.0–34.9)	125.7 (28.7–831.7)
Pregnant women with T18 fetuses	83	55.0 (35.0–75.0)	28.45 (21.2–34.3)	47.8 (9.8–234.2)
Controls with normal fetuses	797	54.5 (36.8–93.0)	28.13 (18.0–34.99)	106.3 (50.5–1136.4)
Amniocentesis group				
High risk of prenatal screening	297	55.0 (39.0–88.0)	29.2 (18.8–35.0)	121.5 (56.0–1136.4)
Ultrasonic abnormality	115	56.0 (40.0–89.0)	28.3 (18.2–35.0)	105.3 (50.5–937.6)
Polyhydramnios	48	56.5 (40.0–89.0)	29.1 (18.2–35.0)	113.4 (50.8–631.2)
Oligohydramnios	11	50.0 (43.0–57.0)	27.3 (18.7–32.4)	82.0 (61.0–215.0)
Bright light in the fetal ventricle	34	59.0 (45.0–76.0)	27.5 (18.7–34.9)	104.2 (50.5–207.0)
Single umbilical artery	22	55.0 (40.0–73.0)	27.4 (20.1–33.5)	103.9 (54.8–937.6)
Karyotype analysis is strongly required due to social factors	49	54.0 (40.0–82.0)	28.4 (18.2–34.3)	98.3 (56.1–801.8)
Non-puncture group				
Peripheral blood karyotype analysis was performed within 2 years old	336	54.0 (36.8–93.0)	27.1 (18.0–35.0)	100.9 (50.5–705.6)

### sHLA-G Enzyme-Linked Immunosorbent Assay (ELISA)

Serum sHLA-G levels were determined using a sHLA-G ELISA kit (sHLA-G ELISA kit, RD194070100R; Exbio, Prague, Czech Republic), according to the manufacturer's protocol.

### Second Trimester Screening

Blood drawn from pregnant women for prenatal screening was used to detect the biochemical markers AFP, uE3, and free β-hCG using time-resolved immunofluorimetry (AutoDELFIA® AFP/Free hCGβ Dual and Unconjugated Estriol, PerkinElmer Life and Analytical Sciences, Turku, Finland). Invasive prenatal diagnostic techniques were performed to confirm the abnormalities when the pregnant woman was at a high risk for abnormalities. All of the babies were followed up until the first year after birth.

### Statistical Analyses

Statistical analysis was performed using the Statistical Package for the Social Sciences (SPSS) software program, version 13.0 (SPSS, Inc., Chicago, IL). The sHLA-G results were expressed as multiples of the median (MoM) of the control group after logarithmic transformation. AFP, uE3, and free β-hCG levels were expressed as MoM of the appropriate gestational control group after logarithmic transformation. Differences in sHLA-G MoM, AFP MoM, uE3 MoM, and free β-hCG MoM were evaluated by Mann-Whitney *U-*test. A Bivariate correlation analysis was used to analyze the relationship of all analytes. A calculation of the risk value using combinations of four analytes, the maternal age risk, and the maternal weight risk was performed according to Gaussian distribution (Reynolds and Penney, 1990; Spencer et al., 1994, 1999). A Logistic regression analysis was used to assess the risk factors for pregnant women with fetuses possessing abnormal chromosomes. A Receiver operating characteristic (ROC) curve analysis was performed to evaluate the power of maternal sHLA-G levels to discriminate fetuses with normal karyotypes from those with chromosomal abnormalities. The cut-off value for the ROC curve analysis was determined by Youden's index (Fluss et al., 2005). Preliminary experimental results for the calculation of sample size showed that the sensitivity and specificity of sHLA-G MoM was 85% and 88%, respectively. The sample size was calculated by software according to the above rates. The minimum number of subjects required was 784, with an allowable error of 0.1 in a two-tailed test with *p* < 0.05. A *p* < 0.05 was considered to be statistically significant.

## Results

### Maternal sHLA-G Levels in Fetuses With Normal and Abnormal Chromosomes

**Figure 2:**
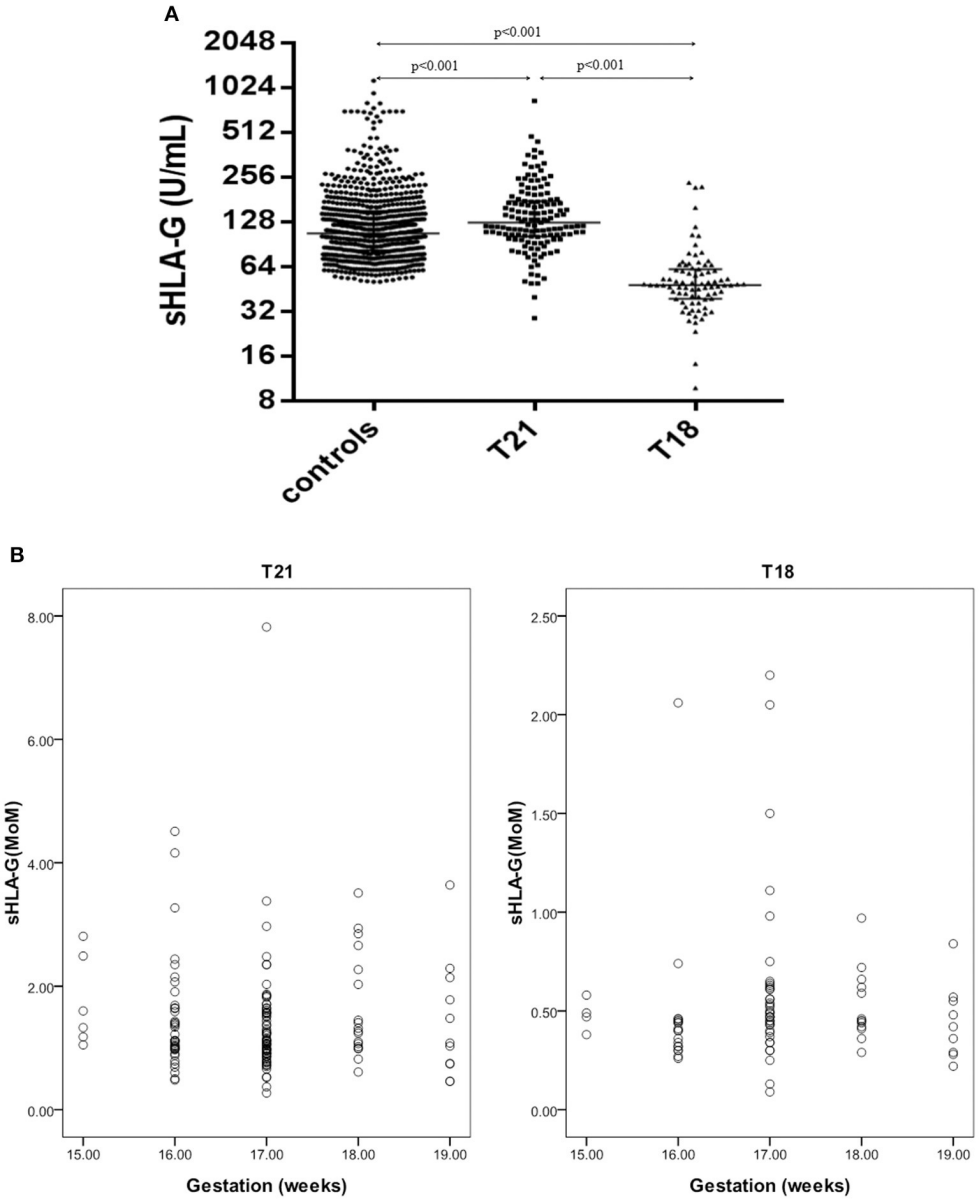
sHLA-G levels and MoM values in pregnant women with T21 fetuses (*n* = 139), T18 fetuses (*n* = 83), and normal control fetuses (*n* = 797). **(A)** Comparison of sHLA-G levels in pregnant women with T21 fetuses, T18 fetuses, and normal control fetuses. **(B)** Scatter diagram of the sHLA-G MoM values of the T21 and T18 groups.

Maternal sHLA-G levels in pregnant women carrying fetuses with normal chromosomes were at a median level of 106.3 U/ml (range: 50.5–1136.4), whereas women carrying T18 fetuses had a median level of 47.8 U/ml (range: 9.8–234.2), and those with T21 fetuses had a median level of 125.7 U/ml (range: 28.7–831.7). Statistical analyses showed that maternal sHLA-G levels in women carrying T21 fetuses were dramatically higher compared to women with normal fetuses (125.7 U/ml vs. 106.3 U/ml; *p* < 0.001), while maternal sHLA-G levels significantly decreased in pregnant women with T18 fetuses (47.8 U/ml vs. 106.3 U/ml; *p* < 0.001) compared to pregnant women with normal chromosome fetuses (Figure 2).

Overall, sHLA-G levels were not associated with the gestational age of the fetus. When the 297 high-risk cases were removed, the median sHLA-G levels first decreased and then increased with the gestational weeks, but there was no statistical difference (data not shown).

### Evaluation of sHLA-G Levels as a Second Trimester Screening Marker in Comparison to Levels of AFP, uE3, and Free β-hCG

To consider variation of marker levels with gestational age, the maternal serum AFP, uE3, and free β-hCG MoM values were used (Spencer et al., 1994, 1999). The AFP, uE3, free β-hCG, and sHLA-G MoM values of the T18 group were remarkably lower than those of the control group (Table 2, all *p* < 0.001). The AFP and uE3 MoM values of the T21 group were statistically lower than the control group, but the free β-hCG and sHLA-G MoM values were noticeably higher than the control group (Table 2, all *p* < 0.001).

**Table 2:** Distribution of data in controls and pregnant women with T21 or T18 fetuses.

	**Controls with normal fetuses**	**Pregnant women with T21 fetuses**	**Pregnant women with T18 fetuses**
	**(*n =* 797)**	**(*n =* 139)**	**(*n =* 83)**
Median AFP MoM	1	0.7149	0.5401
Median free β-hCG MoM	1	2.5804	0.2219
Median UE3 MoM	1	0.7891	0.8330
Median sHLA-G MoM	1	1.1800	0.4500
Mean Log_10_ AFP MoM	−0.0067	−0.1457	−0.1978
Mean Log_10_ free β-hCG MoM	0.0622	0.3651	−0.6133
Mean Log_10_ UE3 MoM	−0.0163	−0.1390	−0.3538
Mean Log_10_ sHLA-G MoM	0.0455	0.1014	−0.3269
SD Log_10_ AFP MoM	0.16057	0.20747	0.25615
SD Log_10_ free β-hCG MoM	0.39822	0.45804	0.52362
SD Log_10_ UE3 MoM	0.16962	0.23251	0.28058
SD Log_10_ sHLA-G MoM	0.22564	0.22433	0.21661
Correlation			
AFP vs. free β-hCG	−0.134 (*P* < 0.01)	0.167 (*P* = 0.05)	0.191 (*P* > 0.05)
AFP vs. UE3	0.525 (*P* < 0.01)	0.228 (*P* < 0.05)	−0.054 (*P* > 0.05)
AFP vs. sHLA-G	−0.075 (*P* < 0.05)	0.003 (*P* > 0.05)	−0.033 (*P* > 0.05)
free β-hCG vs. UE3	−0.293 (*P* < 0.01)	−0.075 (*P* > 0.05)	0.233 (*P* = 0.034)
free β-hCG vs. sHLA-G	0.095 (*P* < 0.01)	0.291 (*P* < 0.01)	−0.030 (*P* > 0.05)
UE3 vs. sHLA-G	−0.052 (*P* > 0.05)	0.164 (*P* > 0.05)	0.212 (*P* > 0.05)

The risk values were calculated by mean and standard deviation (SD) log_10_ analyte MoM (Table 2), then adding the maternal age and weight risk. According to the Gaussian distribution formula, the smaller the numerical values of SD and the greater the distances of the mean between experimental and control groups, the better the analyte for the calculation of the risk values. From the data in Table 2, sHLA-G (T18 group: −0.3269 and 0.21661, control: 0.0455 and 0.22564) had the same predictive value as free β-hCG (T18 group: −0.6133 and 0.52362, control: 0.0622 and 0.39822) for T18 screening. A Bivariate correlation analysis was used to analyze the relationship of all analytes. The results showed that the sHLA-G level was not dependent on AFP, uE3, and free β-hCG in pregnant women with T18 fetuses (Table 2).

### Assessment of the Efficacy of sHLA-G, AFP, uE3, and Free β-hCG as a Marker for Second Trimester Screening Using Logistic Regression

The multivariate logistic regression analyses of age, weight, sHLA-G MoM, AFP MoM, uE3 MoM, and free β-hCG MoM are presented in Table 3. It was found that the sHLA-G MoM [Exp(B): 171.26, 95% CI: 36.30–807.97, *p* < 0.001] and uE3 MoM [Exp(B): 51.11, 95% CI: 16.84–155.11, *p* < 0.001] were risk factors for pregnant women with T18 fetuses.

**Table 3:** The multivariate logistic regression analyses of the various parameters for pregnant women with abnormal chromosome fetuses.

**Variate**	**Pregnant women with T21 fetuses**		**Pregnant women with T18 fetuses**	
	**Exp(B) (95% C-I)**	***p***	**Exp(B) (95% CI)**	***p***
Age	1.02 (0.99–1.04)	0.215	1.01 (0.97–1.06)	0.548
Weight	0.99 (0.94–1.04)	0.574	0.98 (0.90–1.07)	0.701
AFP MoM	6.50 (3.28–12.88)	<0.001	1.60 (0.99–2.59)	0.055
Free β-hCG MoM	0.76 (0.70–0.83)	<0.001	1.30 (0.98–1.72)	0.74
UE3 MoM	3.73 (1.83–7.60)	<0.001	51.11 (16.84–155.11)	<0.001
sHLA-G MoM	0.98 (0.82–1.18)	0.839	171.26 (36.30–807.97)	<0.001

### ROC Analysis for sHLA-G, AFP, uE3, and Free β-hCG as a Screening Marker

**Figure 3:**
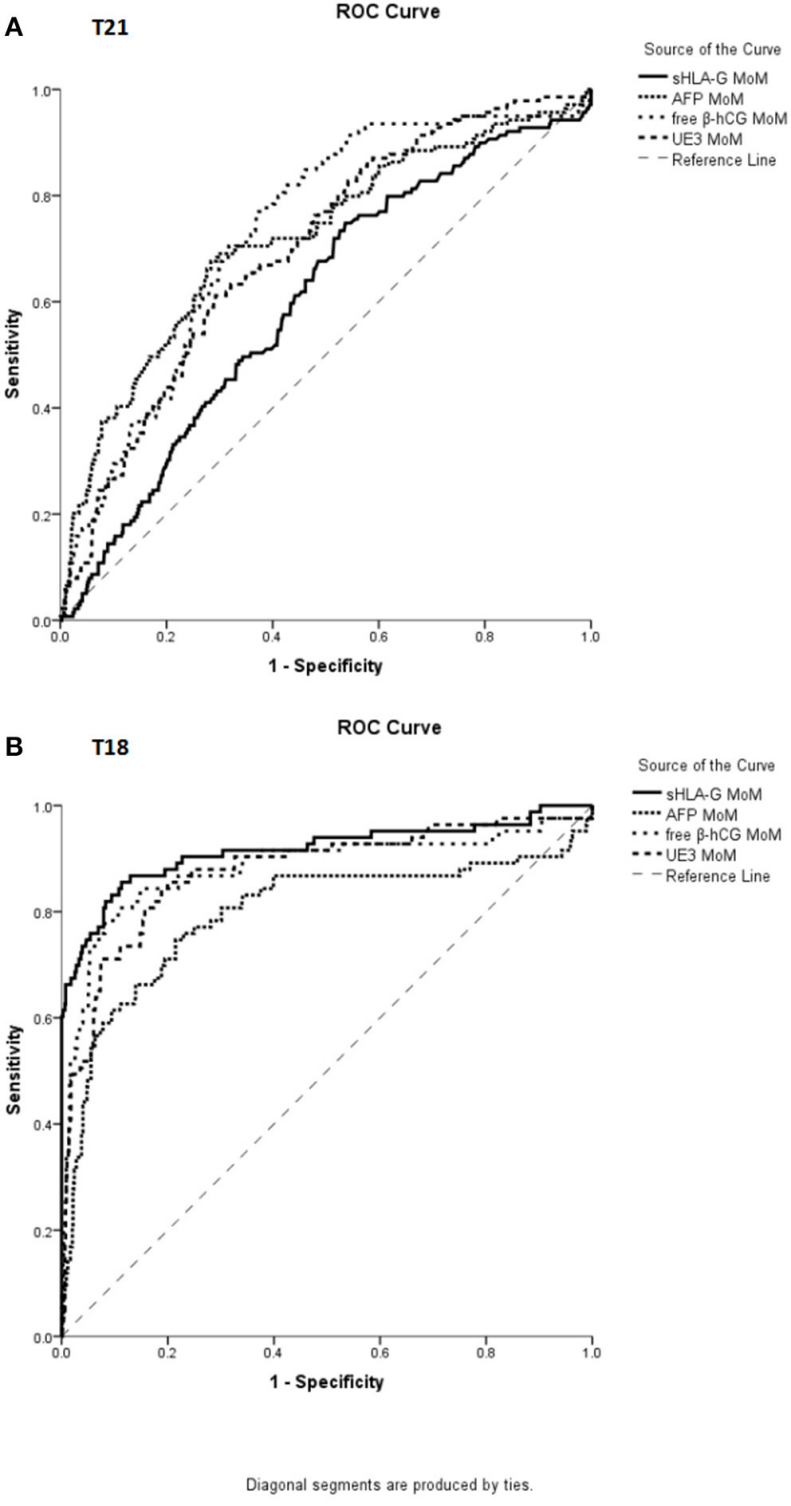
ROC curve analysis for the MoM value of sHLA-G, AFP, free β-hCG, and uE3 in distinguishing pregnant women with normal fetuses from abnormal karyotype fetuses. **(A)** For screening T21, the area under ROC curve for sHLA-G MoM was 0.601 (95% CI, 0.551–0.650, *p* < 0.001), AFP MoM was 0.719 (95% CI, 0.670–0.769, *p* < 0.001), free β-hCG MoM was 0.732 (95% CI, 0.689–0.776, *p* < 0.001), and uE3 MoM was 0.698 (95% CI, 0.653–0.743, *p* < 0.001). **(B)** For screening T18, the area under ROC curve for sHLA-G MoM was 0.915 (95% CI, 0.871–0.959, *p* < 0.001), AFP MoM was 0.796 (95% CI, 0.730–0.861, *p* < 0.001), free β-hCG MoM was 0.881 (95% CI, 0.829–0.934, *p* < 0.001), and uE3 MoM was 0.876 (95% CI, 0.828–0.923, *p* < 0.001).

ROC curves were used to evaluate the performance of four analytes to discriminate pregnant women with normal chromosome fetuses from those with abnormal chromosome fetuses. For the screening of T21 abnormalities, the area under ROC curve for sHLA-G MoM was 0.601 (95% CI, 0.551–0.650, *p* < 0.001), for AFP MoM was 0.719 (95% CI, 0.670–0.769, *p* < 0.001), free β-hCG MoM was 0.732 (95% CI, 0.689–0.776, *p* < 0.001), and uE3 MoM was 0.698 (95% CI, 0.653–0.743, *p* < 0.001) (Figure 3). For the screening of T18 abnormalities, the area under ROC curve for sHLA-G MoM was 0.915 (95% CI, 0.871–0.959, *p* < 0.001), AFP MoM was 0.796 (95% CI, 0.730–0.861, *p* < 0.001), free β-hCG MoM was 0.881 (95% CI, 0.829–0.934, *p* < 0.001), and uE3 MoM was 0.876 (95% CI, 0.828–0.923, *p* < 0.001) (Figure 3).

The cut-off value was determined by Youden's index based on the ROC curve. ROC curve analysis revealed that the MoM value of sHLA-G could powerfully discriminate T18 fetuses with the cut-off <0.646 (sensitivity: 85.5%, specificity: 87.7%; AUC: 0.915; *p* < 0.001) from the normal control group fetuses (Table 4).

**Table 4:** Sensitivity and specificity for the MoM value of sHLA-G, AFP, free β-hCG, and UE3 at specified cutoffs.

**Variables**	**sHLA-G MoM**	**AFP MoM**	**free β-hCG MoM**	**UE3 MoM**
Cutoff	0.646	0.746	0.353	0.76
AUC ± SE	0.915 ± 0.023	0.796 ± 0.034	0.881 ± 0.027	0.876 ± 0.024
(95% CI)	(0.871–0.959)	(0.730–0.861)	(0.829–0.934)	(0.828–0.923)
Sensitivity (%)	85.5%	75.9%	78.3%	84.3%
Specificity (%)	88.7%	78.5%	91.8%	80.1%
PPV (%)	44.1%	26.9%	50.0%	30.6%
NPV (%)	98.3%	96.9%	97.6%	98.0%
PLR	7.58	3.54	9.60	4.23
NLR	0.16	0.31	0.24	0.20
*P*	<0.001	<0.001	<0.001	<0.001

## Discussion

HLA-G was first shown to be expressed on the trophoblast and many studies have since been performed to investigate the role of HLA-G expression in fetal-maternal and reproductive immunology (Kovats et al., 1990; Rajagopalan, 2014). The interactions of HLA-G with its receptors (such as ILT2, ILT4, and KIR2DL4, expressed on uNK cells) have been shown to promote the maintenance of fetal-maternal immunotolerance during pregnancy and have a positive impact on fetal development and growth (Moffett and Colucci, 2015; Ferreira et al., 2017). However, the relationship between maternal peripheral sHLA-G levels and the status of fetal chromosome abnormality was previously unknown. In this study, maternal sera were obtained from women who underwent second trimester prenatal screening, and the chromosomes of fetuses and babies were analyzed by karyotyping. We assessed whether the maternal sHLA-G levels have predictive value for the status of fetal chromosome abnormality. Our data showed that maternal sHLA-G levels in pregnant women with T21 fetuses were significantly higher than in those with normal chromosome fetuses, while maternal sHLA-G levels significantly decreased in pregnant women with T18 fetuses.

Previous studies have revealed that higher levels of sHLA-G in an embryo culture is related to better embryo development, cleavage stages, and to a higher pregnancy rate (Kotze et al., 2013; Diaz et al., 2019). sHLA-G detection has been presented as a non-invasive method for embryo selection and a predictor of pregnancy outcome (Kotze et al., 2010; Rebmann et al., 2010). Based on the sHLA-G levels in the culture supernatant from a 46-h embryo, specific embryos were selected as beneficial to pregnancy and implantation rates, regardless of age (Sher et al., 2004, 2005). In pregnancy disorders, decreased HLA-G expression in extravillous trophoblasts is linked to the incidence of pre-eclampsia. In a cohort of women with placenta-mediated complications of pregnancy (such as pre-eclampsia, fetal growth restriction, stillbirth, and placental abruption), plasma sHLA-G levels were significantly lower at the beginning of pregnancy in these subjects compared with the control group (Marozio et al., 2017). Plasma sHLA-G was also found to be dramatically decreased in women with recurrent abortion compared to normal pregnancies or women with only one abortion (Zidi et al., 2016). A recent study showed that HLA-G expressed on fetal villus tissues was noticeably reduced in trisomy 16-induced early embryonic death (EED) and patients with EED, but normal fetal chromosomes (EEDNC) compared to villous tissues from patients undergoing elective abortion with normal fetal chromosomes (EANC) (Yao et al., 2019).

In our study, maternal sHLA-G levels in pregnant women with T21 fetuses were significantly higher than in those with normal chromosome fetuses, while maternal sHLA-G levels significantly decreased in pregnant women with T18 fetuses. Because it was the first study on pregnancy with chromosomal abnormal fetus and sHLA-G, there was no correlational research to explain the results. Several possible reasons for these results can be speculated. (1) HLA-G was expressed on the trophoblast cell. Arizawa and Nakayama (1992) showed that there was a tendency for heavy placentas in T21, and a tendency for light placentas in T18 and T13. The quantity of trophoblast cell and the placenta size with T18, T21, and normal fetuses might be different (Kennerknecht and Terinde, 1990). These differences might affect the expression and secretion of sHLA-G level. (2) A high sHLA-G level in pregnant women with T21 fetuses might be the reason for their high survival rate.

Prenatal risk screening using maternal serological markers and fetal karyotype diagnosis is an important measure to prevent birth defects and genetic diseases such as the T18 and T21 syndromes. Maternal serological screening using AFP, uE3, and free β-hCG in the second trimester has been widely applied in China and the performance of these markers in prenatal screening has been well-documented (Tu et al., 2016; Zhou et al., 2018). However, other serological markers (such as inhibin) and protocols (such as non-invasive prenatal screening tests (NIPT) and first trimester screening) are also carried out to improve the capacity and quality of prenatal screening (Nshimyumukiza et al., 2018; Breveglieri et al., 2019).

Multiple factors influencing pregnancy were considered in the selection of the control group, but we observed some factors, noted in Table 1, that might have an impact on the sHLA-G level. In order to better compare the screening efficacy of maternal sHLA-G levels, these factors should be considered. Indeed, the need to take these factors into account has inspired our future research directions.

The mean and SD log_10_ analyte MoM values indicated that maternal sHLA-G possessed the same predictive value as free β-hCG in distinguishing the T18 group from the control group, according to Gaussian distribution (Reynolds and Penney, 1990). A Logistic regression analysis showed that sHLA-G MoM, and uE3 MoM were risk factors for pregnant women with T18 fetuses, while ROC analysis revealed that maternal sHLA-G MoM could efficiently discriminate the T18 and T21 fetuses from the normal group. Indeed, the area under ROC curve, sensitivity, and NPV (negative predictive value) of sHLA-G MoM revealed that sHLA-G levels performed better than AFP MoM, free β-hCG MoM, and uE3 MoM in discriminating T18 fetuses from normal fetuses.

Our findings, for the first time, reveal that maternal sHLA-G could be a novel screening marker for T21 and T18 fetuses, particularly for T18. However, a larger cohort is needed to establish a new combined screening formula for clinical use. In addition, the application of sHLA-G in early pregnancy screening is a further research direction of our group.

## Data Availability Statement

The datasets generated for this study are available on request to the corresponding author.

## Ethics Statement

This study was approved by the Ethics Committee of Taizhou Hospital of Zhejiang Province. The patients/participants provided their written informed consent to participate in this study.

## Author Contributions

DX, YZ, and WY contributed to the study design. MD and LG were in charge of specimen selection. LL and YX performed the laboratory work. DX contributed to the analysis of the data and wrote the manuscript. All authors read and approved the final manuscript.

## Conflict of Interest

The authors declare that the research was conducted in the absence of any commercial or financial relationships that could be construed as a potential conflict of interest.
